# Dysregulated low-density granulocyte contributes to early spontaneous abortion

**DOI:** 10.3389/fimmu.2023.1119756

**Published:** 2023-02-23

**Authors:** Hongxia Ye, Lan Li, Yajun Dong, Qu Zheng, Yulin Sha, Li Li, Panyu Yang, Yan Jia, Jiang Gu

**Affiliations:** ^1^ Department of Reproductive Immunology, Chengdu Xi’nan Gynecology Hospital, Chengdu, Sichuan, China; ^2^ Department of Reproductive Immunology, Chengdu Jinjiang Hospital for Maternal & Child Health Care, Chengdu, Sichuan, China; ^3^ Jinxin Research Institute for Reproductive Medicine and Genetics, Chengdu Xi’nan Gynecology Hospital, Chengdu, Sichuan, China; ^4^ Key Laboratory of Transplant Engineering and Immunology, Regenerative Medicine Research Center, West China Hospital, Sichuan University, Chengdu, China; ^5^ Department of Laboratory Medicine, Chengdu Xi’nan Gynecology Hospital, Chengdu, Sichuan, China; ^6^ Department of Gynecology, Sichuan Jinxin Women & Children Hospital, Chengdu, Sichuan, China; ^7^ Department of Pathology and Pathophysiology, Provincial Key Laboratory of Molecular Pathology and Personalized Medicine, Center of Collaborative and Creative Center, Shantou University Medical College, Shantou, China

**Keywords:** low-density granulocytes, normal-density granulocytes, peripheral blood mononuclear cells, maternal-fetal immunity, neutrophil extracellular traps, early-pregnancy, spontaneous abortion

## Abstract

Spontaneous abortion (SA) is a common adverse pregnancy event with unclarified pathogenesis and limited therapeutic efficiency. Although most SA cases with the euploid embryo(s) are associated with immunological factors, the contribution of low-density granulocyte (LDG) in SA pathogenesis is rarely reported. This study aimed to investigate the serial characteristics and possible contribution of LDG and their subpopulations in early pregnancy, especially in early SA. Unpregnant (UP), normally pregnant (NP), and SA women were recruited, and the peripheral blood and endometrium/decidua were collected for LDG isolation and histological observation. The percentage, phenotype, and subpopulations of LDG were analyzed *via* flow cytometric analysis, and the ability of Nets formation was assessed by immunofluorescent and immunohistochemical assays. As a result, 43 participants were enrolled, including 10 UP, 15 NP, and 18 SA women. Compared with the UP group, the LDG percentage in peripheral blood mononuclear cells (PBMCs) and decidual immune cells (DICs) increased in the NP group, while the loss of this increase was observed in the SA group. Meanwhile, CD16^int/−^ cell percentage in peripheral blood LDG (PB-LDG) increased in the NP and SA groups, and insufficient activation of CD16^hi^ PB-LDG characterized by reduced CD11b expression was discovered in the SA group. Moreover, the LDG percentage in DICs was higher than that in PBMCs, and the decidual LDG (D-LDG) showed a surface marker expression profile that is easier to be activated in the pregnant cohort (NP + SA women). Finally, increased decidual Nets formation was observed in the SA group compared with the NP group, and more Nets formation was detected in D-LDG of NP and SA women following PMA stimulation. Overall, LDG participates in the maintenance of early pregnancy, while dysregulated LDG is responsible for early SA, providing novel potential targets for further exploration of SA pathogenesis and therapeutics.

## Introduction

From the perspective of immunology, the embryo can be considered a semi-homograft, carrying half of the genes of both parents and interacting with the maternal immune system without being rejected (1). Spontaneous abortion (SA) is a common adverse pregnancy event, but the etiology is not well described, and therapeutic efficiency is also far from satisfactory. Growing evidence suggests that most SA cases in the euploid embryo(s) are associated with immune factors. Except for the known autoimmune disorders, such as antiphospholipid syndrome and positive anti-thyroid antibodies, the immunological mechanisms of a considerable part of SA are still unclear (2). Meanwhile, the known endocrine, metabolic, and infectious factors, such as polycystic ovarian syndrome, hypothyroidism, and chronic endometritis, could perturb the decidualization process. However, a defect in decidualization can result from changes in immune cells, at least partially (2, 3). Therefore, expanding the understanding of pregnancy-related immune changes and potential mechanisms is urgently needed to explore SA pathogenesis and therapeutic strategies.

Various immune cells infiltrate the maternal-fetal interface in early pregnancy and regulate immune balance. As a critical part of innate immunity, neutrophils are involved in pregnancy and delivery, especially in fertilized egg formation and embryo implantation (4). Neutrophils kill pathogens *via* multiple antimicrobial mechanisms, such as phagocytosis, reactive oxygen species (ROS), and releasing bactericidal enzymes and neutrophil extracellular traps (Nets) (5). Nets are extracellular web-like DNA decorated with histones and antimicrobial proteins released from activated neutrophils and form a powerful antimicrobial mechanism. Nets contain various damage-associated molecular patterns, including DNA, so uncontrolled Nets formation can sustain inflammation and cause tissue damage or dysfunction in the host (6). In addition to immune response, NETs are also involved in angiogenesis, thrombosis, and tissue remodeling (7, 8).

Although the underlying mechanisms are debated, abnormal changes in neutrophil activity are associated with pregnancy complications, including spontaneous abortion (SA) (9, 10). Previous studies have confirmed that neutrophils can be isolated from decidua in the first three months of pregnancy, and decidual neutrophils show an activated phenotype and increased anti-apoptotic ability (9, 11). However, the phenotypes, functional characteristics, and interrelationships among the subtypes of neutrophils in SA, both in peripheral blood (PB) and decidua, have not been clarified. Low-density granulocyte (LDG) were first reported in systemic lupus erythematosus (SLE), and their subsets coexisted with monocytes after gradient centrifugation. Most autoimmune or infectious diseases studies showed that LDG is a group of pro-inflammatory cells. Meanwhile, compared with normal-density neutrophils (NDG), LDG is easier to be activated and exhibits stronger NETosis (12, 13), which reflects the state of neutrophils with NETs formation and contributes to the host defense against pathogens.

LDG is mainly reported in peripheral blood of autoimmune diseases, tumors, infections, thrombosis, and other conditions. Croxatto et al. reported that leukocytes isolated from decidua contained, in the purified mononuclear cell frequency, a population of LDG (11), indicating that LDG may play a role in pregnancy. However, the role and potential mechanisms of LDG in pregnancy and adverse pregnancy events (such as SA) are rarely reported. Therefore, this study aimed to investigate the serial characteristics of LDG, including the percentage, phenotype and subpopulations, and ability to form Nets (Netosis), in peripheral blood and endometrium/decidua of unpregnant, normally pregnant, and SA women and analyze the possible contribution of LDG in early pregnancy and SA, which have not been reported yet. As a result, the findings contributed new evidence of the involvement of LDG in pregnancy and highlighted the participation of dysregulated LDG and its subpopulations in SA pathogenesis, laying a foundation for further study of SA pathogenesis and therapeutics.

## Materials and methods

### Participants

This study was approved by the Reproductive Medicine Ethics Committee of Chengdu Jinjiang Hospital for Maternal & Child Health Care (approval number: 2019003) and followed the Helsinki Declaration. All participants were informed of the nature of the study and signed a written informed consent before participation.

The participants were recruited between Oct 2020 and Mar 2022, including unpregnant (UP), normally pregnant (NP), and SA women. Intrauterine pregnancy was diagnosed through serum and urine β-human chorionic gonadotropin (β-hCG) tests and Doppler ultrasound, and the SA was diagnosed as the unintentional end of pregnancy. The inclusion criteria were: (1) less than 35 years old, body mass index (BMI) < 28 kg/m^2^, and no history of smoking and drinking in the three months before endometrial collection (UP women) or during this pregnancy (NP and SA women); (2) for participants in UP and NP groups, no history of adverse pregnancy (such as SA, premature delivery, pregnancy-induced hypertension, placental abruption), hypertension, cryptorrhea (such as diabetes), and immune and infectious diseases; for participants in SA group, experienced at least one SA before this pregnancy, no normal childbearing history, and no use of immunomodulators and anticoagulants during this pregnancy; (3) no anatomical abnormalities of uterine, such as septate, unicornate, bicornate and didelphis uteri, were observed in the ultrasound examination before participation; (4) the UP volunteers agreed to donate endometrial tissue one week after ovulation, the NP women chose to terminate the healthy pregnancies voluntarily, and the SA women selected induced abortion after the demised fetus was confirmed; (5) no chromosomal abnormalities were identified in the aborted tissues; (6) the days of pregnancy (DOP) in NP and SA women were 42 to 70 days. As a result, 43 participants were recruited, including 10, 15, and 18 in the UP, NP, and SA groups, respectively, and the general characteristics are shown in **Table S1**.

### Collection of peripheral blood and decidual/endometrial tissues

The peripheral blood in all groups was collected. A part was used for blood routine and D-dimer tests, a part was used for plasma separation and dsDNA level determination, and the rest was used to separate peripheral blood mononuclear cells (PBMCs) and normal-density granulocyte (NDG).

For NP and SA participants, fetal heartbeat was confirmed again with a Doppler ultrasound before the induced abortion. After vacuum aspiration, the endometrial/decidual tissues were picked out from the aspirated tissues and repeatedly washed with normal saline to minimize blood contamination. The non-decidual tissue was carefully examined and removed. Part of the endometrial/decidual tissues was fixed and paraffin-embedded for immunofluorescent and immunohistochemical staining, and the remainder was stored in a culture medium for isolation of endometrial/decidual immune cells (EICs/DICs).

### Isolation of PBMCs and EICs/DICs

PBMCs were isolated with Histopaque-1119 and Histopaque-1077 (Sigma, USA) gradient centrifugation procedure. Briefly, Histopaque-1119, Histopaque-1077, and whole blood samples were sequentially added into a 50ml centrifuge tube and centrifuged at 700×g for 30 mins at room temperature. The stratified liquid from top to bottom is plasma, PBMCs (including LDG), Histopaque-1077, peripheral blood NDG (PB-NDG), Histopaque-1119, and red blood cells. The PBMCs and PB-NDG were collected, and the former was used for subsequent peripheral blood LDG (PB-LDG) screening and isolation.

Endometrial and decidual tissues were cut into about 1 mm^3^ pieces and digested with 1 mg/mL (0.1%) collagenase type IV (Sigma, USA) and 150 U/ml DNase I (Sigma) at 37°C for about 60 mins with gentle agitation. The cell suspension was passed through 100μm, 70μm, and 40μm cell strainer and centrifuged at 700×g in a discontinuous Percoll gradient (20%, 40%, and 60%) for 30 mins. The decidual immune cells (DICs) between 40% and 60% Percoll solution (densities of 1.056 to 1.077 g/mL) were collected and used for subsequent endometrial LDG (E-LDG) and decidual LDG (D-LDG) isolation.

### Phenotypic analysis of PBMCs, PB-NDG, and EICs/DICs by flow cytometry

The isolated PBMCs and E/DICs were washed with stain buffer and subsequently stained with the antibody-conjugates, including APC-Cy7 CD45, PerCP-Cy5.5 CD15, BV510 CD14, BV711 CD62L, BV605 CD11b/MAC-1, FITC CD16, and Alexa 647 CD66b (BD, USA), for 30 minutes at 4°C in the dark. PB-NDG were stained with the above antibodies except for APC-Cy7 CD45, PerCP-Cy5.5 CD15, and BV510 CD14 because the concentration of PB-DNG with CD45^+^CD15^+^CD14^-^ we separated was more than 97% (**Figure S1A**). Then, the stained cells were washed with stain buffer and acquired *via* an FCM with CellQuest software. More than 1 × 10^4^ cells in each sample were detected. The results were analyzed using Flowjo software and expressed as a percentage of positive cells.

### LDG isolation and identification

LDG were isolated from the PBMCs and E/DICs by magnetic bead selection and identified by FCM. Briefly, the PBMCs and E/DICs were incubated with anti-CD14 mAbs (Milteny, Germany) for 15 mins, and the CD14- cells were isolated by negative selection using anti-CD14 magnetic beads and MACS dissociator (Milteny). The CD14- cells were further incubated with anti-CD15 mAbs (Milteny) for 15 mins, and the CD15+/CD14- cells (LDG) were isolated by positive selection using anti-CD15 magnetic beads and MACS dissociator. The purity of the isolated LDG was identified by staining the cells for 30 min at 4°C with monoclonal antibodies specific for CD14 and CD15 and evaluating them by FCM. The percentage of CD14-/CD15+ cells was identified as more than 95%.

### Quantitation of NTEs *via* dsDNA detection

dsDNA in NETs of plasma was quantified by Picogreen dsDNA Assay kit (Invitrogen, USA) according to the manufacturer’s instructions.

### Immunofluorescent and immunohistochemical staining

For immunofluorescent staining, paraffin slides of endometrial/decidual tissues were processed and incubated with primary antibodies, including anti-CD15 (Abcam, USA; 1:1000) and anti-H3Cit (ab5103, USA; 1:1000), before being stained with fluorescent secondary antibodies and DAPI. The images were acquired with a fluorescence microscope (Olympus, Japan), and the number of endometrial/decidual samples with Nets positive was counted. Meanwhile, the paraffin slides were processed and stained with PAD4 antibody (Affinity, USA; 1:200) before being stained with secondary antibodies and chromogen substrate for immunohistochemical staining.

### Phorbol-12-myristate-13-acetate stimulation and Nets formation detection

The isolated PB-LDG, PB-NDG, and E/D-LDG were seeded on a poly-lysine-pretreated glass cover in a 48-well plate and stimulated with PMA (100 ng/mL, Sigma) for 4 hours. Then, the cells were fixed with 4% paraformaldehyde (Sigma) and incubated with primary antibodies, including anti-MPO (Abcam, USA; 1:1000) and anti-H3cit (Abcam; 1:1000), followed by staining with fluorescent secondary antibodies and DAPI. Images were obtained using a FluoView FV1000 confocal microscope (Olympus, Japan) and analyzed with Olympus FV10-ASW software (Olympus). The percentage of positively stained cells was calculated, and all sections were assessed independently by three blinded investigators (Ye Hx, Lan Li, and Jia Y), and the quantitative results were determined *via* consensus.

### Statistical analysis

Statistical analyses and data graphs were generated with GraphPad Prism software (version 9.4, GraphPad Inc., USA). Continuous data were expressed as median with interquartile range (IQR) and assessed for normality using the Kolmogorov-Smirnov and D’Agostino & Pearson tests. Student’s *t*-test was used to analyze the differences between the two groups. When the variances of the two groups differed in the *F* test, the Mann-Whitney *U*-test was used to compare the two groups. One-way analysis of variance with Tukey’s *post hoc* analysis (for normally distributed data) or Kruskal-Wallis *H* with Student-Newman-Keuls analysis (for non-normally distributed data) was performed for comparisons among three or more groups. A *P* < 0.05 was considered statistically significant.

## Results

### General characteristics of the enrolled participants

Forty-three participants were enrolled in this study, including 10 unpregnant (UP), 15 normally pregnant (NP), and 18 SA women (**Table S1** and **S2**). There was no significant difference in age and body mass index (BMI) among the three groups and in days of pregnancy (DOP) between the NP and SA groups. Meanwhile, all participants in the SA group experienced at least one SA before this pregnancy (at least two SA if this SA was included) (**Table S1**). Meanwhile, there was no significant difference in the peripheral blood neutrophils/lymphocytes ratio (PB-NLR), platelet/lymphocytes ratio (PB-PLR), and D-dimer levels among the three groups (**Table S3**).

### LDG percentage in PBMCs increases after eight weeks of normal pregnancy, while the loss of this increase is associated with SA

**Figure 1:**
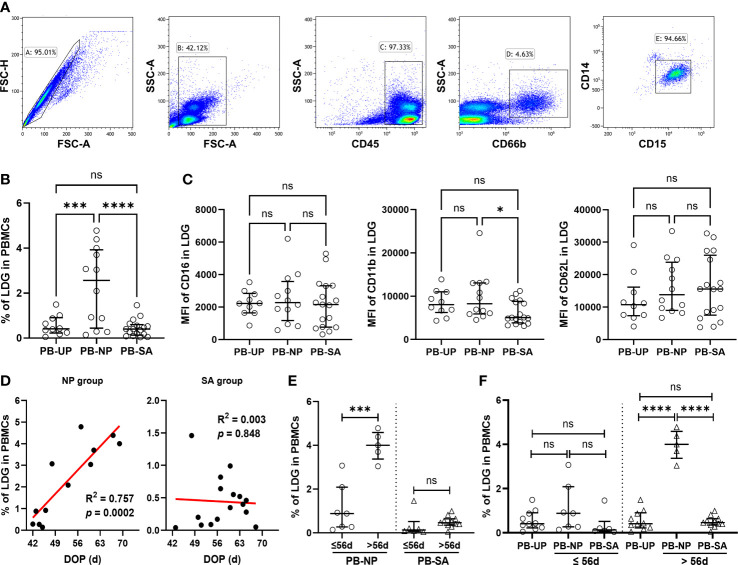
Loss of increase and insufficient activation in PB-LDG is associated with SA. PBMCs were isolated from the peripheral blood of UP, NP, and SA women by density gradient centrifugation. **(A)** PB-LDG was identified as SSC^hi^CD45^+^CD15^+^CD14^-^ singlets according to the gating strategy shown in the five panels. **(B)** Comparison of the LDG percentage in PBMCs of 10 UP, 12 NP, and 17 SA women. **(C)** Comparison of the fluorescence intensity (MFI) of CD16, CD11b, and CD62L in PB-LDG among UP, NP, and SA groups. **(D)** Correlation analysis of the LDG percentage in PBMCs and DOP in pregnant women (NP + SA). Data are presented as dots and median with interquartile range, and each symbol represents an individual donor. **(E)** Comparison of the LDG percentage in PBMCs between the subgroup with DOP > 56d or ≤ 56d in NP and SA groups. **(F)** Comparison of the LDG percentage in PBMCs among UP, NP, and SA groups with DOP ≤ 56d or > 56d. T-test or Mann-Whitney U test was used to identify the differences between the two groups, and one-way analysis of variance (ANOVA) with Tukey’s *post hoc* analysis or Kruskal-Wallis H with Student-Newman-Keuls *post hoc* analysis was performed for comparisons among the three groups. * p < 0.05, *** p < 0.001, **** p < 0.0001. ns, not significant.

In PBMCs, the LDG percentage was quantified *via* FCM analysis (**Figure 1A**), and the difference among the groups was investigated. As shown in **Figure 1B**, the LDG percentage was significantly higher in the NP group than in the UP and SA groups, indicating that the LDG percentage in PBMCs increases in normal pregnancy, and the loss of this increase may be associated with SA. Meanwhile, no significant difference in the MFI of CD16 and CD62L was observed among the three groups, but the MFI of CD11b was significantly higher in the NP group than in the SA group, suggesting an insufficient activation of PB-LDG in the SA group (**Figure 1C**).

Interestingly, correlation analysis revealed that the LDG percentage in PBMCs was positively correlated with DOP in the NP group but was not in the SA group (**Figure 1D**). Then, subgroup analysis was performed according to whether the DOP was more than 56d (when the placenta began to form and take over the function of the corpus luteum at about eight weeks). The LDG percentage in the NP subgroup with DOP > 56d (57-70 days) was significantly higher than that in the subgroup with DOP ≤ 56d (42-56 days). However, subgroup difference was not observed in the SA group (**Figure 1E**). Meanwhile, in women with DOP ≤ 56d, the LDG percentage showed no significant difference among the three groups. However, in women with DOP > 56d, the LDG percentage in the NP group was significantly higher than in the UP and SA groups (**Figure 1F**). These findings indicate that the LDG percentage in PBMCs increases mainly after eight weeks of normal pregnancy, and the loss of this increase may also be related to SA.

### Increased CD16^int/−^ PB-LDG correlates with pregnancy, and insufficient activation of the CD16^hi^ PB-LDG may be associated with SA

**Figure 2:**
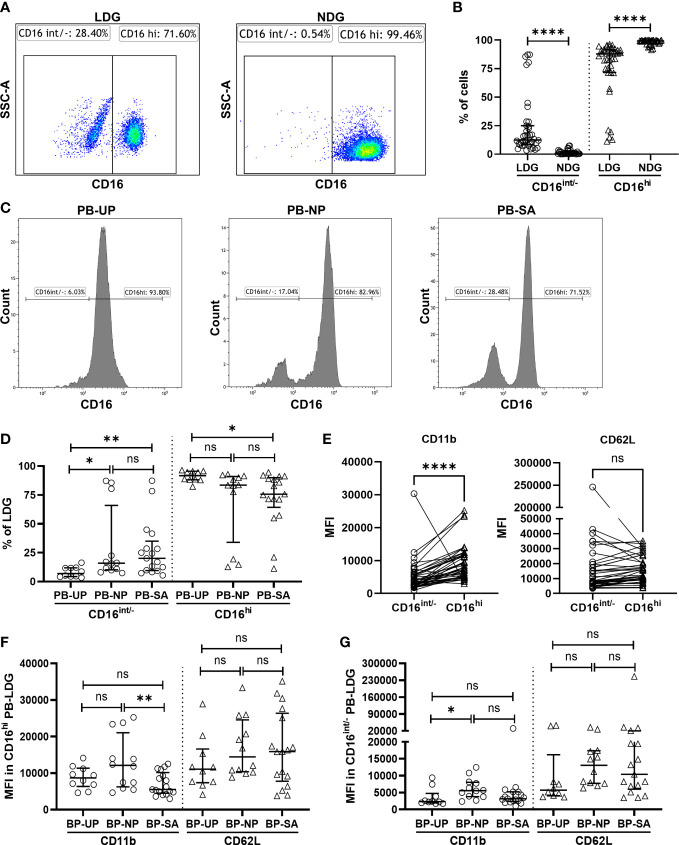
Insufficient activation of decreased CD16^hi^ PB-LDG correlates with SA. **(A)** PB-LDG and PB-NDG were classified as CD16^int/-^ and CD16^hi^ subpopulations and data were shown from representative CD16 plots in FCM analysis. **(B)** Comparison of the percentage of CD16^int/-^ and CD16^hi^ cells between PB-LDG and PB-NDG. **(C)** Representative FCM analysis of the percentage of CD16^int/−^ and CD16^hi^ cells in PB-LDG. **(D)** Comparison of the percentage of CD16^int/−^ and CD16^hi^ cells in PB-LDG. **(E)** Comparison of the MFI of CD11b and CD62L between CD16^int/-^ and CD16^hi^ LDG. **(F, G)** Comparison of the MFI of CD11b and CD62L in CD16^hi^ LDG **(F)** and CD16 ^int/-^ LDG **(G)**. Data are presented as dots and median with interquartile range, and each symbol represents an individual donor. T-test or Mann-Whitney U test was used to identify the differences between the two groups, and one-way ANOVA with Tukey’s *post hoc* analysis or Kruskal-Wallis H with Student-Newman-Keuls *post hoc* analysis was performed for comparisons among the three groups. * p < 0.05, ** p < 0.01, **** p < 0.0001. ns, not significant.

According to the CD16 expression, peripheral blood NDG (PB-NDG) and PB-LDG can be classified as CD16^int/-^ and CD16^hi^ subpopulations and representative CD16 plots in FCM analysis are shown in **Figure 2A**. In PB-NDG, no significant difference was observed in the percentage of CD16hi and CD16int/- cells and in the mean fluorescence intensity (MFI) of CD62L and CD11b among the three groups (**Table S4** and **Figure S1**). The percentage of CD16^int/-^ cells was significantly higher, while the percentage of CD16^hi^ cells was significantly lower in LDG than in NDG (**Figure 2B**). In PB-LDG, the percentage of CD16^int/−^ cells in the NP and SA groups was significantly higher, while CD16^hi^ cells in the SA group were significantly lower than those in the UP group. However, there was no significant difference in the CD16^int/−^ and CD16^hi^ cell percentage between the NP and SA groups (**Figures 2C, D**). These results suggest that increased CD16^int/−^ PB-LDG is related to pregnancy, while a decreased CD16^hi^ PB-LDG may be associated with SA.

The immune cell surface markers were further analyzed to explore the potential link between PB-LDG and SA. As shown in **Figure 2E**, the MFI of CD11b in CD16^hi^ LDG was significantly higher than that in CD16^int/-^ LDG, suggesting that CD16^hi^ LDG is more easily activated than CD16^int/-^ subsets. In CD16^hi^ LDG, the MFI of CD11b in the SA group was significantly lower than that in the NP group (**Figure 2F**), while this difference was not observed in CD16^int/-^ LDG (**Figure 2G**). These results suggest an insufficient activation of CD16^hi^ LDG in the SA group, and this was further supported by the findings that the MFI of CD11b of CD16^hi^ LDG in UP and NP groups was more than twice that of CD16^int/-^ LDG (mean: 8762.18 vs. 3555.96 and 12942.11 vs. 6196.477, respectively) but less than 50% higher in the SA group (mean: 5051.374 vs. 7005.47) (**Table S5**). Therefore, the above results indicate that the insufficient activation of the CD16^hi^ PB-LDG may be related to SA.

### LDG percentage in DICs increases in normal pregnancy, and the loss of this increase is associated with SA

**Figure 3:**
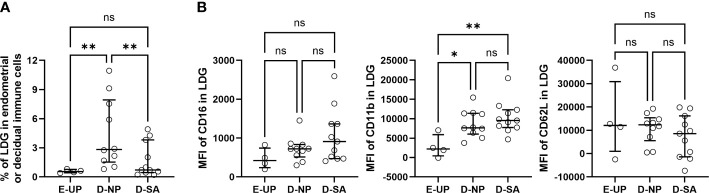
Loss of increase in decidual LDG (D-LDG) is associated with SA. Endometrial/decidual immune cells (EICs/DICs) were isolated, and the LDG percentage in EICs/DICs was quantified and identified. **(A)** Comparison of the LDG percentage in EICs/DICs of 4 UP, 10 NP, and 11 SA women. **(B)** Comparison of the MFI of CD16, CD11b, and CD62L in E/D-LDG among UP, NP, and SA groups. Data are presented as dots and median with interquartile range, and each symbol represents an individual donor. One-way ANOVA with Tukey’s *post hoc* analysis or Kruskal-Wallis H with Student-Newman-Keuls *post hoc* analysis was performed for comparisons among the three groups. * p < 0.05, ** p < 0.01. ns, not significant.

EICs/DICs with a density of less than 1.077 mg/ml were isolated, and the LDG percentage in EICs/DICs was quantified. The results were consistent with that in PBMCs, i.e., the NP group was significantly higher than SA and UP groups (**Figure 3A**), indicating the LDG percentage in DICs increases in normal pregnancy, and the loss of this increase is associated with SA. Meanwhile, the expressions of CD16, CD11b, and CD62L in E/D-LDG were also analyzed. No significant difference in the MFI of CD16 and CD62L among the three groups was observed, but the MFI of CD11b in the NP and SA groups was significantly higher than that in the UP group, suggesting increased activation of D-LDG in early pregnancy (**Figure 3B**). Although the MFI of CD11b in the SA group was higher than in the NP group, the difference was not statistically significant.

### LDG percentage is higher in DICs than in PBMCs, and D-LDG is more easily activated than PB-LDG in early pregnancy

**Figure 4:**
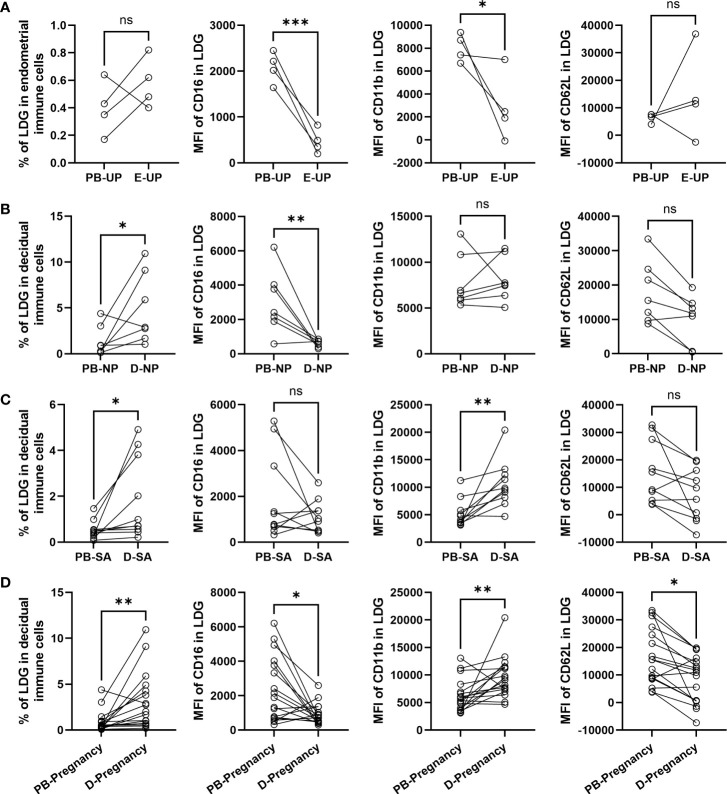
LDG percentage is higher in DICs than in PBMCs, and D-LDG is more easily activated than PB-LDG in early pregnancy. **(A-D)** The LDG percentage in PBMCs and EICs/DICs and the MFI of CD16, CD11b, and CD62L between PB-LDG and E/D-LDG in the UP **(A)**, NP **(B)**, and SA **(C)**, and pregnant (NP +SA) **(D)** women were quantified and compared. Data are presented as dots, and each symbol represents an individual donor. T-test or Mann-Whitney U test was used to identify the differences between the two groups. * p < 0.05, ** p < 0.01, *** p < 0.001. ns, not significant.

The LDG percentage in PBMCs and EICs/DICs and the surface markers in PB-LDG and E/D-LDG were compared. There was no statistical difference in the LDG percentage between PBMCs and EICs in the UP group (**Figure 4A**), but a significantly higher LDG percentage in DICs than in PBMCs was observed in the NP and SA groups (**Figures 4B, C**). Compared with PB-LDG, the MFI of CD16 in UP and NP groups and CD11b in the UP group was significantly lower, while the MFI of CD11b in the SA group was significantly higher in E/D-LDG (**Figures 4A-C**). Moreover, in the pregnant women (NP + SA), there were significantly higher LDG percentage in DICs and MFI of CD11b in D-LDG and significantly lower MFI of CD16 and CD62L in D-LDG than in PBMCs and PB-LDG, respectively (**Figure 4D**). These results indicate that LDG percentage is higher in DICs than in PBMCs, and D-LDG is more easily activated than PB-LDG in early pregnancy.

### Increased decidual Nets formation is associated with SA

**Figure 5:**
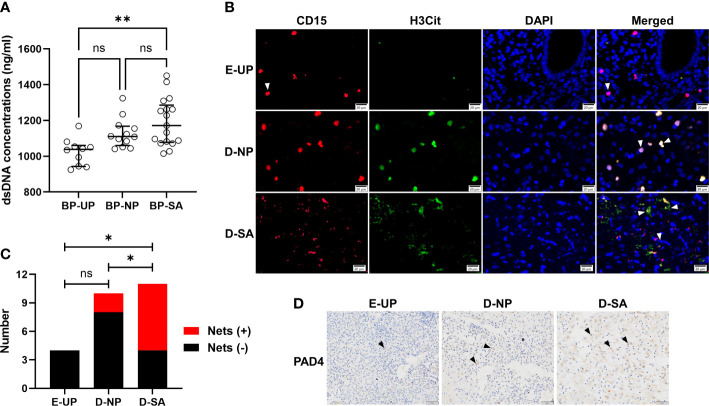
Increased decidual Nets formation is associated with SA. **(A)** Comparison of the dsDNA concentration in peripheral blood plasma among UP, NP, and SA groups. **(B, C)** The Nets formation in the endometrial/decidual tissues was double-labeled, in which neutrophils were labeled with CD15 (red) and Nets were labeled with H3cit (green) **(B)**, and the number of Nets-positive decidual/endometrial samples was counted and compared among UP, NP, and SA groups **(C)**. The white arrows indicated typical neutrophil changes in different groups. **(D)** Representative image of the immunohistochemical staining of PAD4 (black arrows), which was associated with Nets formation, in endometrial/decidual tissues. Data are presented as dots and median with interquartile range (**A**, each symbol represents an individual donor) or case number **(C)**. One-way ANOVA with Tukey’s *post hoc* analysis or Kruskal-Wallis H with Student-Newman-Keuls *post hoc* analysis was performed for comparisons among the three groups. * p < 0.05, ** p < 0.01. ns, not significant.

Peripheral blood plasma was collected, and the dsDNA concentration was detected to determine the formation of Nets. The results showed that the dsDNA concentration in UP, NP, and SA groups increased gradually, and the highest value was observed in the SA group and was significantly higher than in the UP group (**Figure 5A**). Meanwhile, the Nets formation in the endometrial/decidual tissues was investigated with double-label immunofluorescence, in which neutrophils were labeled with CD15 (red) and Nets were labeled with H3cit (green). As shown in **Figure 5B**, CD15-positive neutrophils in the UP group were morphologically intact with little H3cit expression. The CD15-positive neutrophils were also intact in the NP group, but H3cit increased without prominent reticular structure, suggesting a restrained Nets formation. In the SA group, the CD15-positive neutrophils were fragmentary, and H3cit appeared in a reticular pattern, indicating a large amount of depolymerized chromatin and NETs formation. Quantitatively, the number of Nets-positive decidual/endometrial samples (women) was significantly higher in the SA group (7, 58.33%) than in the NP (2, 20%) and UP (0) groups, while there was no significant difference between the NP and UP groups (**Figure 5C**), indicating an increased decidual Nets formation is associated with SA. Because Nets formation was associated with PAD4, immunohistochemical staining also revealed that the PAD4-positive cells in the SA group were overwhelmingly higher than those in the NP and UP groups (**Figure 5D**).

### Increased Nets formation in D-LDG is correlated with early pregnancy

**Figure 6:**
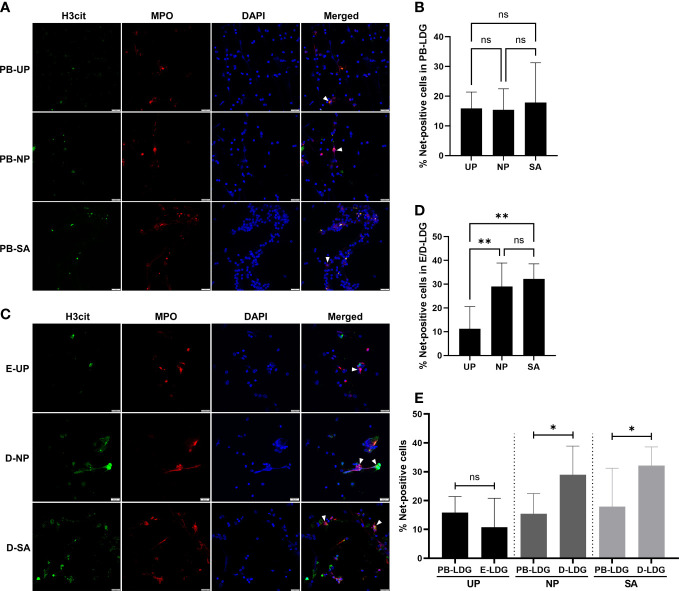
Increased Nets formation in D-LDG is correlated with early pregnancy. The PB-LDG and E/D-LDG were isolated and stimulated by PMA, and the Nets formation was detected and quantified. **(A)** Representative image of Nets formation labeled with H3cit (green) and MPO expression (red) in PB-LDG. **(B)** Comparison of the percentage of Nets-positive cells in PB-LDG. **(C)** Representative image of Nets formation labeled with H3cit (green) and MPO expression (red) in E/D-LDG. **(D)** Comparison of the percentage of Nets-positive cells in E/D-LDG. **(E)** Comparison of the percentage of Nets-positive cells between PB-LDG and E/D-LDG. Data are presented as median with interquartile range. T-test or Mann-Whitney U test was used to identify the differences between the two groups, and one-way ANOVA with Tukey’s *post hoc* analysis or Kruskal-Wallis H with Student-Newman-Keuls *post hoc* analysis was performed for comparisons among the three groups. * p < 0.05, ** p < 0.01. ns, not significant.

To further clarify whether the Nets formation in PB-LDG and D-LDG was related to pregnancy or SA, the PB-NDG, PB-LDG, and E/D-LDG were isolated and stimulated by PMA, and the Nets formation was quantified. As a result, Nets formation was detected in all cells following PMA stimulation. There was no statistical difference in the percentage of Nets-positive cells in PB-LDG and PB-NDG among the three groups (**Figures 6A, B** and **S2A**, **S2B**) and in the percentage of Nets-positive cells between PB-LDG and PB-NDG (**Figure S2C**). However, the Nets formation in D-LDG of the SA and NP groups was significantly higher than that in E-LDG of the UP group (**Figures 6C, D**). Meanwhile, the percentage of Nets-positive cells in the SA and NP groups was significantly higher in D-LDG than in PB-LDG, while no significant difference in the UP group (**Figure 6E**). These results indicate that both PB-NDG, PB-LDG, and E/D-LDG can form Nets, and increased Nets formation in D-LDG is correlated with early pregnancy.

## Discussion

The excessive maternal immune responses must be strictly controlled to ensure a successful pregnancy (14). Although many studies reported the roles of immune cells in maternal-fetal immunity (15), the contribution of neutrophils is often overlooked. In recent years, the involvement of neutrophils and their subpopulations, especially the LDG, in pregnancy is being discovered, but it is still rarely reported in SA (16–18). In this study, we first observed that LDG percentage in PBMCs and DICs increases in normal pregnancy, while the loss of this increase is associated with SA. Meanwhile, we found that the increased CD16^int/−^ cell percentage in PB-LDG correlates with pregnancy, and insufficient activation of the CD16^hi^ PB-LDG may be related to SA. Moreover, the LDG percentage was higher in DICs than in PBMCs, and D-LDG was more easily activated than PB-LDG in early pregnancy. Finally, the histological analysis discovered that increased decidual Nets formation is associated with SA, and the PMA-stimulative assay verified that increased Nets formation in D-LDG correlates with early pregnancy. These findings demonstrated the serial characteristics of LDG and their subpopulations in peripheral blood and endometrium/decidua of unpregnant, normally pregnant, and SA women and highlighted the involvement of LDG in early pregnancy and SA, providing promising targets for further exploration of SA pathogenesis and therapeutics.

The neutrophil is a homogeneous and terminally differentiated cell population with higher density than mononuclear cells because of the enrichment of granular proteins (19). Recent studies identified several neutrophil subpopulations, of which LDG attracts increasing attention (20–22). However, the definition of granulocyte with lower density is not unified. Except for LDG, many other terms are also commonly used, such as low-density neutrophils (LDN) (6, 23), granulocytic myeloid-derived suppressor cells (G-MDSC) (24, 25), polymorphonuclear myeloid-derived suppressor cells (PMN-MDSC) (26–28). Of note, the definitions and functions are reported differently for these cells. For example, PMN-MDSC and LDN are enriched in the low-density PBMCs fraction following density centrifugation and express similar surface markers, including CD11b^+^CD14^−^CD15^+^CD66b^+^ (23). Unlike these phenotypes, Li et al. defined the PMN-MDSC as HLA-DR^−^/^low^CD11b^+^CD33^+^CD15^+^CD14^−^ (26). Meanwhile, the ability of PMN-MDSC to suppress T cell function was contrary to that of LDN in SLE (23, 29). Moreover, these cells have been reported in other autoimmune, cancer, and infectious diseases with either pro-inflammatory (LDG) or suppressive effects (PMN-MDSC) (22). Overall, these cells may be at least partially identical according to the isolation protocol and phenotype identification. Therefore, it is necessary to conduct more studies on this specific subpopulation of neutrophils to achieve unification of definition and identification.

Compared with the UP women, we found that the LDG percentage in the NP group increased in both peripheral blood and decidua. However, this increase was not observed in SA women. These results are similar to those reported by Li et al. on the changes in PMN-MDSC during early pregnancy (26). In addition, they reported that PMN-MDSC was the main subset of MDSC in human decidua and exhibited an immunosuppressive effect. Our study also confirmed that the LDG percentage in decidua was higher than in PBMCs during early pregnancy. Similar changes in PMN-MDSC were also reported previously (30). Meanwhile, we found that the LDG percentage in PBMCs is associated with gestational age and significantly increased after eight weeks in a normal pregnancy but exhibited inconspicuous changes in SA. However, verifying whether a similar phenomenon exists in D-LDG requires a more extensive sample size. Moreover, the placenta begins to form and take over the function of the corpus luteum in a process termed luteoplacental shift at about eight weeks of pregnancy (2, 31), whether the increase of LDG percentage in PBMCs is a prerequisite for the normal placental formation and function and the maintenance of normal pregnancy is interesting and worth further exploration.

CD16 expresses on many immune cell surfaces and appears late during neutrophilic maturation (32–34). LDG is a mixed population of mature and immature neutrophils and can be classified into different subsets according to the CD16 expression. In adult anti-neutrophil cytoplasm autoantibody vasculitis (AAV) patients and healthy controls, Ui Mhaonaigh et al. revealed that the CD16^+^ cell percentage was significantly lower in LDG than in NDG, and there was a significant increase in CD16^int/−^ cells in the LDG compared to NDG fraction. Our study revealed similar changes in CD16^hi^ and CD16^int/-^ LDG from UP, NP, and SA women compared to NDG. However, the changes of CD16 expression in PB-LDG were like that observed in the LDG in umbilical cord blood and granulocyte of humanized mice treated with granulocyte-colony stimulating factor (G-CSF) (33). Considering that the increase of CD16^int/−^ in LDG may be the result of the acute granulopoiesis-induced increase in the number of immature neutrophils (35), whether the changes of LDG and their subpopulations were a non-specific feature of acute illness need to be verified in more disease models. Nonetheless, we revealed that the percentage of CD16^hi^ LDG in SA women was significantly lower than in UP women, but no significant difference was observed between NP and UP women. Therefore, whether a decreased CD16^hi^ PB-LDG is associated with SA needs more clarification.

Integrin CD11b is a receptor expressed on various leukocytes, such as monocytes, neutrophils, dendritic cells, and NK cells (36, 37). After granulocyte activation, CD11b transfers from an intracellular pool to the external surface of the neutrophil plasma and plays various biological functions, such as host defense, cellular inflammatory responses, and signal transduction (38, 39). CD11b exhibits anti-inflammatory effects, and recent studies have shown that CD11b activation inhibits TLR-dependent inflammation and autoimmunity, thereby reducing inflammatory damage (40). Meanwhile, CD11b deficient mice showed susceptibility to inflammatory and autoimmune diseases (41). In sepsis and SLE models, CD11b deficiency led to increased levels of pro-inflammatory cytokines (42). Sacks et al. reported that the expression of CD11b on the surface of leukocytes in NP women was higher than that in UP women (43). Our study revealed similar results in neutrophils, but CD11b expression in SA women was significantly lower than in NP women, indicating that the decreased CD11b expression might affect the immune response in SA pathogenesis.

Nets are reticular structures mainly composed of citrullinated histones, myeloperoxidase, neutrophil elastase, and other components and are involved in multifarious physical and pathological conditions (44–46). This study discovered that the Nets formation during pregnancy increased in peripheral blood and decidua, and this is consistent with the view that human pregnancy is associated with a mild pro-inflammatory state characterized by circulatory neutrophil activation (47). Nets formation during pregnancy was regulated in a multi-mode manner, such as HCG and CSF could promote NETosis, while progesterone restrained the NETotic process (48, 49). In the mouse model of early pregnancy, abundant Nets formation at the maternal-fetal interface could lead to fetal death (14). This study also found that the Nets formation in SA decidua was higher than that in NP decidua, indicating that excessive Nets formation may be responsible for fetal death in early SA. Moreover, PAD4 deficiency could lead to a failure of Nets formation and a significant reduction of pregnancy loss (50). We also found that the PAD4-positive cells in the SA decidua were overwhelmingly higher than those in the NP and UP decidua, indicating that PAD4 may be a crucial intersection between the Nets formation and SA occurrence and represents a promising therapeutic target.

This study also has several limitations. Firstly, this study was carried out in a small-sized human sample, and studies with a large ample are still needed. Secondly, SA is a highly heterogeneous disease with complex and diverse etiology. Some maternal risk factors, such as chronic endometritis, endocrine abnormalities, and antiphospholipid syndrome, were not considered in the inclusion criteria, and the relevant subgroup analysis was also not conducted due to the small sample size. Thirdly, DOP-based subgroup analysis was not performed in E/D-LDG because not enough decidual tissues with DOP > 56d were collected. Finally, endometrial and decidual NDG was not isolated for study, and CD16-based subgroup analysis in E/D-LDG was not conducted because the subpopulations could not be distinguished *via* FCM analysis.

In conclusion, LDG in peripheral blood and decidua participates in the maintenance of early pregnancy, while dysregulated LDG, involving the percentage, phenotype and subpopulations, and ability to form Nets, is responsible for early SA. Of course, more studies with larger sample sizes, optimized functional and rescue assays, and animal experiments, even human trials, are still required in the subsequent exploration of LDG-based immunological mechanisms and therapeutic strategies of SA.

## Data availability statement

The original contributions presented in the study are included in the article/**Supplementary Material**. Further inquiries can be directed to the corresponding authors.

## Ethics statement

The studies involving human participants were reviewed and approved by Chengdu Xi’nan Gynecology Hospital. The patients/participants provided their written informed consent to participate in this study.

## Author contributions

HY conceived the project and designed experiments. HY, YD, LiL, and YJ screened and collected human tissues. HY, LaL, QZ, YS, and PY performed the experiments and acquired, analyzed, and interpreted the data. HY wrote the manuscript. YJ and JG supervised the project. All authors contributed to the article and approved the submitted version.
