# Supplementary material for: Dysregulated low-density granulocyte contributes to early spontaneous abortion
Source: Front Immunol. 2023 Feb 23;14:1119756. doi: 10.3389/fimmu.2023.1119756 (PMC9995479; doi:10.3389/fimmu.2023.1119756)
Supplement: Supplementary file 1 [file DataSheet_1.docx]

**Supplemental materials**

**TABLE S1** General characteristics of the included participants

| Characteristics | UP group | NP group | SA group | *Statistics* | *P* |
| --- | --- | --- | --- | --- | --- |
| No. | 10 | 15 | 18 | - | - |
| Age (years) | 31.00 (25.75-33.25) | 30.00 (29.00-34.00) | 31.00 (26.75-34.00) | 0.106 | 0.900 |
| BMI (kg/m^2^） | 21.44 (20.03-22.35) | 20.70 (20.02-23.43) | 23.01 (21.30-24.23) | 2.997 | 0.061 |
| No. of previous SA | 0 | 0 | 2.00 (1.00-2.00) | - | - |
| DOP (d) | - | 53.00 (44.00-60.00) | 58.5 (53.25-64) | 1.863 | 0.072 |
| *Abbreviations:* UP, unpregnant; NP, normal pregnant; SA, spontaneous abortion; BMI, body mass index; SA, spontaneous abortion; DOP, days of pregnancy.  Continuous variables were expressed as median (interquartile range), and a *P* < 0.05 was considered statistically significant. | | | | | |

**TABLE S2** General characteristics of the participants in the SA group

| **No.** | **Genetic factors^1^** | **Anatomical factors^2^** | **Autoimmune factors** | |  | **Prethromboticstate** | |  | **Endocrine factors** | | |  | **Infectious factors** | **Smoking & Alcohol abuse** |
| --- | --- | --- | --- | --- | --- | --- | --- | --- | --- | --- | --- | --- | --- | --- |
|  |  |  | APA | ATA |  | Congenital | Acquired |  | Thyroid dysfunction^3^ | PCOS | LPD |  | TORCH |  |
| 1 | No | No | No | No |  | No | No |  | No | No | No |  | No | No |
| 2 | No | No | No | No |  | No | No |  | No | No | No |  | No | No |
| 3 | No | No | No | No |  | No | No |  | No | No | No |  | No | No |
| 4 | No | No | ***Yes*** | No |  | No | ***Yes*** |  | No | ***Yes*** | No |  | No | No |
| 5 | No | No | No | No |  | No | No |  | No | No | No |  | No | No |
| 6 | No | No | No | No |  | No | No |  | No | No | No |  | No | No |
| 7 | No | No | No | No |  | No | No |  | No | No | No |  | No | No |
| 8 | No | No | ***Yes*** | No |  | No | ***Yes*** |  | No | No | No |  | No | No |
| 9 | No | No | No | No |  | No | No |  | No | No | No |  | No | No |
| 10 | No | No | ***Yes*** | No |  | No | ***Yes*** |  | No | No | No |  | No | No |
| 11 | No | No | No | No |  | No | No |  | No | ***Yes*** | No |  | No | No |
| 12 | No | No | No | No |  | No | No |  | No | No | No |  | No | No |
| 13 | No | No | No | No |  | No | No |  | No | No | No |  | No | No |
| 14 | No | No | No | No |  | No | No |  | No | No | No |  | No | No |
| 15 | No | No | No | No |  | No | No |  | No | No | No |  | No | No |
| 16 | No | No | No | No |  | No | No |  | No | No | No |  | No | No |
| 17 | No | No | No | No |  | No | No |  | No | No | No |  | No | No |
| 18 | No | No | No | No |  | No | No |  | No | No | No |  | No | No |
| ***Notes:*** ^1^, Both embryonic and parental chromosomal abnormalities were excluded; ^2^, Both congenital and acquired uterine abnormalities were excluded; ^3^, Overt hypothyroidism or hyperthyroidism.  ***Abbreviations:*** APA, antiphospholipid antibody; ATA, anti-thyroid antibodies; PCOS, polycystic ovarian syndrome; LPD, luteal phase defects. | | | | | | | | | | | | | | |

**TABLE S3** Comparisons of the peripheral blood neutrophils/lymphocytes ratio, platelet/lymphocytes ratio, and D-dimer level among the groups

|  | UP group | NP group | SA group | *Statistics* | *P* |
| --- | --- | --- | --- | --- | --- |
| N | 10 | 13 | 17 | - | - |
| Age (years) | 31.00 (25.75-33.25) | 30.00 (28.50-33.50) | 31.00 (26.50-34.00) | 0.052 | 0.950 |
| BMI (kg/m^2^） | 21.44 (20.03-22.35) | 20.44 (20.02-22.77) | 22.86 (21.30-24.24) | 2.792 | 0.074 |
| No. of previous SA | 0 | 0 | 2.00 (1.00-2.00) | - | - |
| DOP (d) | - | 53.00 (44.00-60.50) | 60.00 (52.50-64.00) | 1.648 | 0.111 |
| PB-NLR | 3.00 (2.44-3.90) | 2.92 (2.25-3.43) | 3.50 (2.89-5.04) | 5.538 | 0.063 |
| PB-PLR | 155.12 (123.40-168.31) | 137.61 (102.10-155.35) | 120.39 (107.98-149.25) | 2.512 | 0.285 |
| D-dimer (mg/ml) | 0.17 (0.14-0.23) | 0.19 (0.16-0.27) | 0.21 (0.19-0.25) | 1.467 | 0.244 |
| *Abbreviations:* UP, unpregnant; NP, normal pregnant; SA, spontaneous abortion; BMI, body mass index; SA, spontaneous abortion; DOP, days of pregnancy; PB-NLR, peripheral blood neutrophils/lymphocytes ratio; PB-PLR, peripheral blood platelet/lymphocytes ratio.  Continuous variables were expressed as median (interquartile range), and a *P* < 0.05 was considered statistically significant. | | | | | |

**TABLE S4** Comparisons of the subpopulations and immune surface markers in peripheral blood normal-density neutrophils (PB-NDG)

|  | UP group | NP group | SA group | *Statistics* | *P* |
| --- | --- | --- | --- | --- | --- |
| N | 10 | 11 | 15 | - | - |
| Age (years) | 31.00 (25.75-33.25) | 30.00 (28.00-33.00) | 31.00 (26.00-34.00) | 0.021 | 0.980 |
| BMI (kg/m^2^） | 21.44 (20.03-22.35) | 20.95 (20.02-23.43) | 22.86 (21.3-24.26) | 2.321 | 0.114 |
| No. of previous SA | 0 | 0 | 2.00 (1.00-2.00) | - | - |
| DOP (d) | - | 53.00 (45.00-61.00) | 60.00 (54.00-64.00) | 1.528 | 0.140 |
| CD16^hi^ frequency | 99.45 (98.71-99.89) | 99.38 (95.97-99.73) | 98.81 (95.72-99.56) | 2.068 | 0.356 |
| CD16^int/-^ frequency | 0.70 (0.30-1.18) | 0.48 (0.24-3.59) | 1.06 (0.40-3.15) | 3.132 | 0.209 |
| CD62L MFI | 702.82 (453.07-897.01) | 924.36 (650.07-1202.54) | 636.90 (361.10-2252.00) | 2.093 | 0.351 |
| CD11b MFI | 429.27 (314.52-634.25) | 644.00 (426.01-1202.36) | 384.90 (198.70-650.70) | 3.070 | 0.060 |
| *Abbreviations:* UP, unpregnant; NP, normal pregnant; SA, spontaneous abortion; BMI, body mass index; SA, spontaneous abortion; DOP, days of pregnancy.  Continuous variables were expressed as median (interquartile range), and a *P* < 0.05 was considered statistically significant. | | | | | |

**TABLE S5** Comparisons of the immune surface markers in the subpopulations of peripheral blood low-density neutrophils (PB-LDG)

| Group | Sub-group | N | Mean fluorescence intensity (MFI) | *Statistics* | *P* |
| --- | --- | --- | --- | --- | --- |
| UP group |  |  |  |  |  |
| CD62L | CD16^int/-^ | 10 | 5715.88 (4058.66-16209.16) | 30.000 | 0.143 |
|  | CD16^hi^ | 10 | 11073.44 (7377.97-16579.35) |  |  |
| CD11b | CD16^int/-^ | 10 | 2309.94 (1925.61-4749.95) | 9.000 | 0.001 |
|  | CD16^hi^ | 10 | 8682.45 (6359.25-11351.20) |  |  |
| NP group |  |  |  |  |  |
| CD62 | CD16^int/-^ | 12 | 13045.86 (7742.73-17379.69) | 0.714 | 0.483 |
|  | CD16^hi^ | 12 | 14462.50 (10394.50-24544.00) |  |  |
| CD11b | CD16^int/-^ | 12 | 5583.91 (3814.61-8062.38) | 2.883 | 0.009 |
|  | CD16^hi^ | 12 | 12107.65 (6248.09-21034.78) |  |  |
| SA group |  |  |  |  |  |
| CD62 | CD16^int/-^ | 17 | 10403.11 (6012.74-20310.45) | 125.500 | 0.523 |
|  | CD16^hi^ | 17 | 15866.72 (7796.64-26396.30) |  |  |
| CD11b | CD16^int/-^ | 17 | 3169.71 (2400.95-5198.85) | 58.000 | 0.002 |
|  | CD16^hi^ | 17 | 5518.27 (4506.89-10148.15) |  |  |
| *Abbreviations:* UP, unpregnant; NP, normal pregnant; SA, spontaneous abortion.  Continuous variables were expressed as median (interquartile range), and a *P* < 0.05 was considered statistically significant. | | | | | |


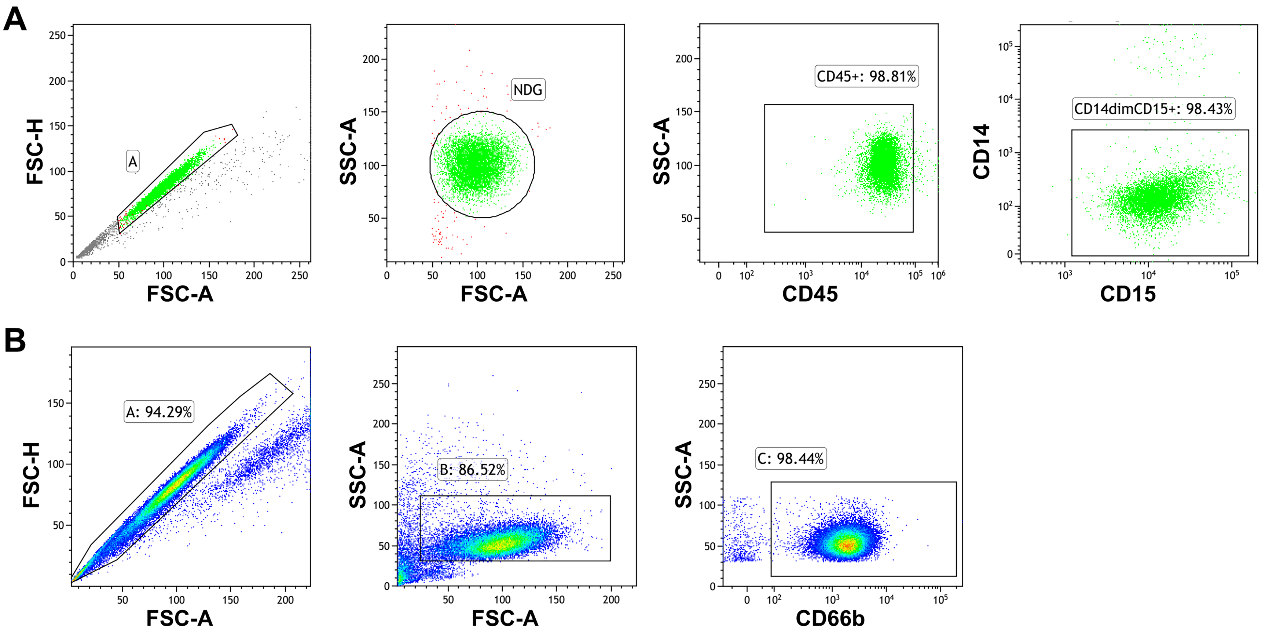


**FIGURE S1**

Identification and quantification of peripheral blood normal-density granulocytes (PB-NDG). **(A)** The frequency of SSC^hi^CD45^+^CD15^+^CD14^-^ in the NDG subgroup (data shown from a representative patient). **(B)** PB-NDG was identified according to the gating strategy shown in the three panels.


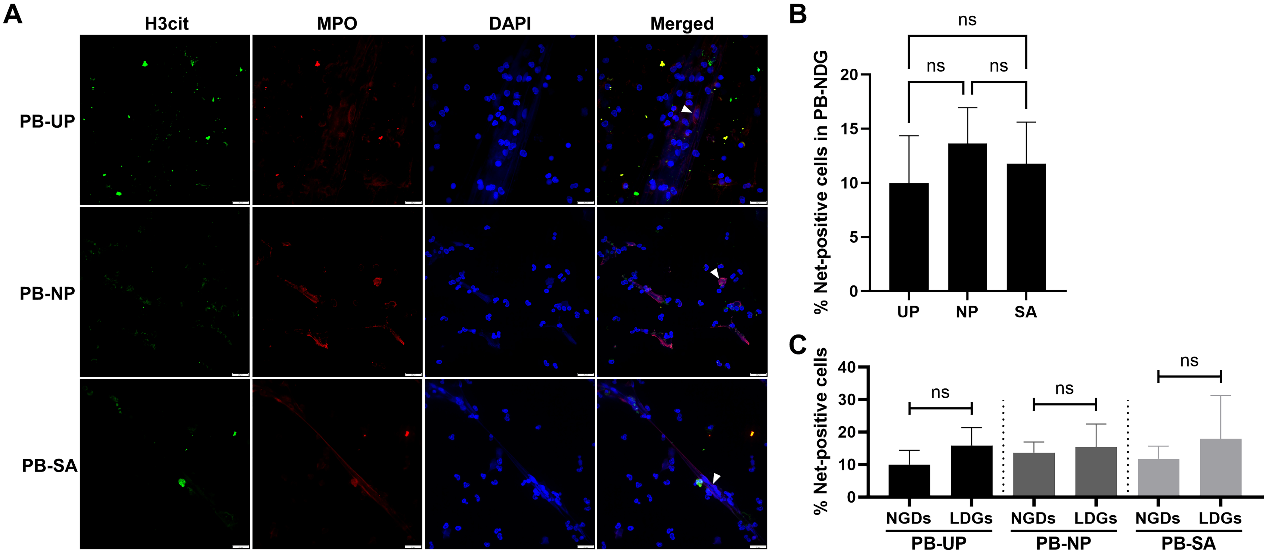


**FIGURE S2**

Nets formation in PB-NDG. The PB-NDG was isolated and stimulated by PMA, and the Nets formation was detected and quantified. **(A)** Representative image of Nets formation labeled with H3cit (green) and MPO expression (red) in PB-NDG. **(B)** Comparison of the percentage of Nets-positive cells in PB-NDG. **(C)** Comparison of the percentage of Nets-positive cells between PB-NDG and PB-LDG. Data are presented as median with interquartile range. T-test or Mann-Whitney U test was used to identify the differences between the two groups, and one-way ANOVA with Tukey’s post hoc analysis or Kruskal-Wallis H with Student-Newman-Keuls post hoc analysis was performed for comparisons among the three groups.
